# Direct Current Stimulation Disrupts Endothelial Glycocalyx and Tight Junctions of the Blood-Brain Barrier *in vitro*

**DOI:** 10.3389/fcell.2021.731028

**Published:** 2021-09-28

**Authors:** Yifan Xia, Yunfei Li, Wasem Khalid, Marom Bikson, Bingmei M. Fu

**Affiliations:** Department of Biomedical Engineering, The City College of the City University of New York, New York, NY, United States

**Keywords:** solute permeability, trans-endothelial electrical resistance (TEER), heparan sulfate, ZO-1, inhibition of endothelial nitric oxide synthase (eNOS), human cerebral microvascular endothelial cells, mouse brain microvascular endothelial cells, hyaluronic acid

## Abstract

Transcranial direct current stimulation (tDCS) is a non-invasive physical therapy to treat many psychiatric disorders and to enhance memory and cognition in healthy individuals. Our recent studies showed that tDCS with the proper dosage and duration can transiently enhance the permeability (P) of the blood-brain barrier (BBB) in rat brain to various sized solutes. Based on the *in vivo* permeability data, a transport model for the paracellular pathway of the BBB also predicted that tDCS can transiently disrupt the endothelial glycocalyx (EG) and the tight junction between endothelial cells. To confirm these predictions and to investigate the structural mechanisms by which tDCS modulates P of the BBB, we directly quantified the EG and tight junctions of *in vitro* BBB models after DCS treatment. Human cerebral microvascular endothelial cells (hCMECs) and mouse brain microvascular endothelial cells (bEnd3) were cultured on the Transwell filter with 3 μm pores to generate *in vitro* BBBs. After confluence, 0.1–1 mA/cm^2^ DCS was applied for 5 and 10 min. TEER and P to dextran-70k of the *in vitro* BBB were measured, HS (heparan sulfate) and hyaluronic acid (HA) of EG was immuno-stained and quantified, as well as the tight junction ZO-1. We found disrupted EG and ZO-1 when P to dextran-70k was increased and TEER was decreased by the DCS. To further investigate the cellular signaling mechanism of DCS on the BBB permeability, we pretreated the *in vitro* BBB with a nitric oxide synthase (NOS) inhibitor, L-NMMA. L-NMMA diminished the effect of DCS on the BBB permeability by protecting the EG and reinforcing tight junctions. These *in vitro* results conform to the *in vivo* observations and confirm the model prediction that DCS can disrupt the EG and tight junction of the BBB. Nevertheless, the *in vivo* effects of DCS are transient which backup its safety in the clinical application. In conclusion, our current study directly elucidates the structural and signaling mechanisms by which DCS modulates the BBB permeability.

## Introduction

As a non-invasive neuromodulation technique, transcranial direct current stimulation (tDCS) has been used to treat various neurological and psychiatric disorders since nineteenth century (Steinberg, 2013; Kekic et al., 2016; Palm et al., 2016; Truong and Bikson, 2018). Weak direct currents across the human brain are introduced via electrodes distributed on the skull, the electric current can alter the brain function by modulating neuron firing rates and changing neuron membrane potentials (Nitsche and Paulus, 2000; Jackson et al., 2016; Woods et al., 2016; Antal et al., 2017; Giordano et al., 2017; Kenney-Jung et al., 2019). In addition to priming neuronal capacity, tDCS has been found to enhance blood perfusion in humans (Stagg et al., 2013; Wang et al., 2015) and in animals (Mielke et al., 2013), and to increase blood nitric oxide (NO) levels (Marceglia et al., 2016).

To safeguard the brain from the blood-borne toxins in the circulating blood, the wall of the cerebral microvessels is specified as the blood-brain barrier (BBB) due to its very low permeability compared to the wall of peripheral microvessels in non-brain tissues. In addition to the endothelium, the BBB consists of basement membrane (BM) filled with extracellular matrix (ECM) and wrapped by the pericytes and astrocyte foot processes (Farkas and Luiten, 2001; Abbott et al., 2006; Fu, 2018). The BBB permeability to water and hydrophilic solutes is determined by its structural components in the paracellular pathway: the endothelial glycocalyx (EG) (Yoon et al., 2017; Kutuzov et al., 2018), the gap space and tight junctions between adjacent endothelial cells (ECs), the width of the BM, the ECM and the gap between astrocyte foot processes (Li et al., 2010b; Li and Fu, 2011; Fu, 2018). Recent study by Shin et al. (2020) showed that tDCS transiently increases the BBB solute permeability in rat brain through activation of nitric oxide synthase (NOS). By employing a transport model for the paracellular pathway of the BBB (Li et al., 2010b), Shin et al. (2020) also predicted that the structural mechanisms by which the tDCS transiently enhances the BBB permeability are temporarily disrupting the EG and the ECM of the BM, disrupting the tight junctions between ECs, as well as increasing the gap width between ECs and that of BM.

The first objective of this study is to confirm the above prediction for the structural mechanisms of tDCS *in vivo*. Since in the BBB, only EG and ECM carry charge, if they are disrupted by the tDCS, the BBB permeability to the same sized solutes with opposite charge should become identical. We thus used the same method and under tDCS with the same strength as in Shin et al. (2020) to determine the rat BBB permeability to FITC-ribonuclease and FITC-α-lactalbumin, which have almost the same size (Stokes radius ≈ 2 nm) but opposite charge (net charge of +4 and −10, respectively) (Yuan et al., 2010b).

Because it is very challenging to directly observe the transiently disrupted EG and tight junctions of the BBB *in vivo*, the second objective is thus to generate *in vitro* BBB models and determine the changes in the EG and tight junctions after tDCS treatments. Human cerebral microvascular endothelial cells (hCMECs) and mouse brain microvascular endothelial cells (bEnd3) were cultured on the Transwell filter with 3 μm pores to generate the *in vitro* BBB. We used similar set up and DCS strength as in Cancel et al. (2018) to treat the *in vitro* BBB. The BBB permeability to ions, the transendothelial electrical resistance (TEER), was measured before and after DCS treatments. Disruption of tight junctions of ECs would significantly affect TEER which is an indicator for permeability to ions or small molecules (Fu et al., 1994; Sugihara-Seki and Fu, 2005). The EG of the *in vitro* BBB formed by hCMEC/bEnd3 cells and the tight junction ZO-1 of that formed by bEnd3 were quantified under control and after DCS treatment. We also quantified *in vitro* BBB permeability to Dex-70k since disruption of EG would significantly affect the BBB permeability to large solutes such as Dex-70k (Fu and Shen, 2003; Sugihara-Seki and Fu, 2005; Yuan et al., 2010a; Kutuzov et al., 2018).

As shown in Shin et al. (2020), tDCS-increased BBB permeability is NO dependent but how is unanswered. Therefore, the third objective of this study is to directly demonstrate how the EG and tight junction ZO-1 of *in vitro* BBB are affected by a NO inhibitor, L-NMMA, a NO donor, SNP, and pretreatment of L-NMMA before DCS. Correspondingly, we also quantified the changes in the BBB TEER and permeability to Dex-70k by these factors. Taken together, our results directly elucidate the structural and signaling mechanisms by which DCS modulates the BBB permeability.

## Materials and Methods

### Solutions and Fluorescent Test Solutes

#### Mammalian Ringer’s Solution and Reagents

Mammalian Ringer’s solution was used for all the permeability measurement, which is composed of (in mM) NaCl 132, KCl 4.6, MgSO_4_ 1.2, CaCl_2_ 2.0, NaHCO_3_ 5.0, glucose 5.5, and HEPES 20. These chemicals were from Sigma-Aldrich. The pH was adjusted to 7.4–7.45 by adjusting the ratio of HEPES acid to base. In addition, the florescent dye solution contained 10 mg/mL BSA (A4378; Sigma-Aldrich, United States) or 1% BSA to maintain the same oncotic pressure as in the plasma (Fu and Shen, 2004). N^*G*^-monomethyl-L-arginine (L-NMMA) and sodium nitroprusside (SNP) were purchased from Sigma (Sigma-Aldrich). L-NMMA (1 mM) and SNP (300 μM) used in the experiments were achieved by dilutions of the stock with 1% BSA-Ringer solution (Zhang et al., 2016). The solutions were made fresh on the day of use to avoid binding to the serum albumin (Adamson et al., 1994; Fu et al., 1998).

#### FITC-Ribonuclease, FITC-a-Lactalbumin and FITC-Dextran-70k

Ribonuclease A (R5500, MW = 13, 683, Sigma-Aldrich) and α-lactalbumin (L6010, MW = 14,176, Sigma-Aldrich) were labeled with fluorescein isothiocyanate isomer I (FITC, F7250, MW = 389.4, Sigma-Aldrich) as described in Adamson et al. (1994); Fu et al. (1998), and Yuan et al. (2010a). The intensity of free dye is less than 1% compared to that of the solution prepared by using this protocol (Yuan et al., 2010a). The influence of the free dye on measured permeability to a labeled protein was discussed by Fu et al. (1998) and contributed less than 0.5% to the BBB permeability for the understudied solutes (Yuan et al., 2010a,b; Shi et al., 2014b). After FITC labeling, the final charge of FITC-ribonuclease is +4 and FITC-α-lactalbumin is −10 (Yuan et al., 2010b; Li and Fu, 2011). The final concentrations FITC-ribonuclease and FITC-α-lactalbumin used in the *in vivo* experiment were 1 mg/mL in the Ringer solution containing 10 mg/mL BSA. In the *in vitro* permeability measurement, the concentration of FITC-ribonuclease, FITC-α-lactalbumin, and FITC-Dextran-70k (FD70s, Sigma-Aldrich) was 10 μM. The concentration of the solution for each solute was chosen to be in the linear range of the concentration vs. fluorescence intensity calibrated in Li et al. (2010b) and Shi et al. (2014b). All dye solutions described above were made fresh on the day of use and discarded at the end of the day.

### *In vivo* Experiments

#### Animal Preparation

All animal care and preparation procedures were approved by the Animal Care and Use Committee at the City College of the City University of New York and all experiments were performed in accordance with relevant guidelines and regulations. All experiments were performed on adult female SD rats (250–300g, Hilltop, Scottdale, PA). The preparation of the rat skull observation area was the same as previously described (Yuan et al., 2009; Shi et al., 2014b; Shin et al., 2020). Briefly, after anesthesia, a section of ∼6 mm by ∼4 mm area (ROI) on the frontoparietal bone was carefully ground with a high-speed micro-grinder (DLT 50KBU, Brasseler, Savannah, GA) until soft and translucent. After grinding, the carotid artery on the same side of the ROI was cannulated with a PE50 tubing (BD Medical, NJ). The rat was then fixed on a stereotaxic alignment system (SAS 597, David Kopf Instruments, Tujunga, CA). After tDCS treatment, the rat head was quickly placed to the objective lens of the multiphoton system for the permeability measurement. It took about 5 min to find the ROI with proper microvessels in the rat brain parenchyma. The images of a cerebral microvessel and its surrounding brain tissue were observed and collected under the objective lens of a multiphoton microscope through the thinned part of the skull.

#### Transcranial Direct Current Stimulation

tDCS was administered using a constant current stimulator (1x1 tDCS, Soterix Medical Inc., New York, United States) to deliver a 1 mA current (the current density 8.0 mA/cm^2^) for 20 min. This dose and duration were the optimal and also safe for the *in vivo* tDCS treatment on rats (Jackson et al., 2017; Shin et al., 2020; Xia et al., 2020). Current was applied transcranially to the frontal cortex of a rat head (approximately 2 mm anterior to Bregma and 2 mm right to Sagittal suture) to obtain similar physiological outcomes as in the human tDCS application studies (Marceglia et al., 2016; Jackson et al., 2017; Shin et al., 2020; Xia et al., 2020). Specifically, an epi-cranial anode (1 mm diameter, Ag/AgCl) in a plastic cannula (4 mm inner diameter) filled with conductive electrolyte gel (Signa, Parker Laboratory, NJ) was positioned onto the rat skull with the location described above. The returning electrode (5 × 5 cm adhesive conductive fabric electrode, AxelGaard, Fallbrook, CA) was placed onto the ventral thoracic region of the anesthetized rat (Shin et al., 2020).

#### Multiphoton Microscopy and Image Analysis

The 12-bit images were collected *in vivo* by a two-photon microscopic system (Ultima, Prarie Technologies Inc., Middleton, WI) with a 40 × lens (NA = 0.8, water immersion, Olympus). The excitation wavelength was set to 820 nm to observe the cerebral microvessels 100–200 μm below the pia mater. The images were taken simultaneously while the fluorescence solution was introduced into the cerebral circulation via the ipsilateral carotid artery at 3 mL/min. We selected a brain region containing post-capillary venules of 20–40 μm diameter and collected images for a ROI of ∼240 μm × 240 μm at a rate of ∼1 frame/s for ∼1 min every 5 min for 20 min (Shin et al., 2020). The images were analyzed off-line using the Image J (National Institutes of Health) to determine the BBB permeability to solutes.

#### Determination of the Blood-Brain Barrier Solute Permeability *in vivo*

The same method as in our previous studies (Shi et al., 2014a; Shin et al., 2020) was used to determine the permeability (P) of the cerebral microvessels in rat brain. P was determined by using the following equation:


$$P=\frac{1}{△I_{0}}\times {\left(\frac{dI}{dt}\right)}_{0}\times \frac{r}{2}$$

Here ΔI_0_ is the step increase of the florescence intensity in the measuring window when the dye just fills up the vessel lumen, (dI/dt)_0_ is the slope of the increasing curve of the total intensity I of the measuring window vs. time t when the solute further diffuses into the surrounding tissue, and r is the vessel radius.

### *In vitro* Experiments

#### Cell Culture and Trans-Endothelial Electrical Resistance Measurement

Two cell lines were used to generate *in vitro* BBB, human brain microvascular endothelial cells (hCMEC/D3 or hCMEC) from Millipore Sigma (Burlington, MA) and mouse brain microvascular endothelial cells (bEnd3) from ATCC (Manassas, VA). hCMECs were cultured in EBM-2 MV endothelial cell growth basal Medium (Lonza, Basel, Switzerland), supplemented with 100 U/mL Penicillin-Streptomycin streptomycin (Gibco, Thermo Fisher Scientific, Waltham, MA). bEnd3 was cultured in Dulbecco’s Modified Eagle’s Medium/Nutrient Mixture F-12 Ham (DMEM/F-12), 2 mM L-glutamine,10% fetal bovine serum (FBS, Gibco, Thermo Fisher Scientific, Waltham, MA), and supplemented with 100 U/mL Penicillin-Streptomycin streptomycin (Gibco, Thermo Fisher Scientific, Waltham, MA). Both cell lines were incubated in the humidified atmosphere with 5% CO_2_ at 37°C. To form an *in vitro* human BBB, hCMECs were seeded at 60 k/cm^2^ to a Transwell filter (PET 3.0 μm pore size, Corning, NY) precoated with 50 μg/mL collagen I (Sigma-Aldrich, St. Louis, MO) and cultured for 5–7 days until confluent. To form an *in vitro* mouse BBB, bEnd3 cells were seeded at 60k/cm^2^ to the same type of the Transwell filter precoated with 30 μg/mL fibronectin (Sigma-Aldrich, St. Louis, MO) and cultured for 4–5 days until confluent. Tran-endothelial electrical resistance (TEER) was monitored 3 days after seeding to check the confluency and barrier formation. TEER was measured by EVOM2 epithelial Volt/Ohm meter with the electrode (STX2) (World Precision Instruments, Sarasota, FL). For each sample, the TEER was measured three times. The average of the three measurements is the TEER for that sample. The TEER of a blank Transwell filter with the same cell culture medium was also measured and subtracted from the TEER of the total system to determine the TEER of the *in vitro* BBB (Li et al., 2010a).

#### Direct Current Stimulation on *in vitro* Blood-Brain Barrier

**FIGURE 1:**
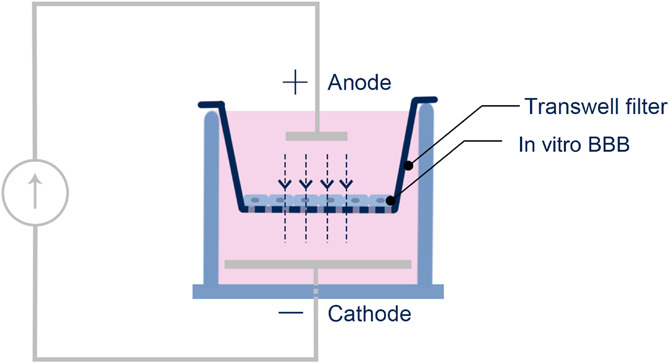
Schematic of the setup for the DCS application to the *in vitro* BBB. The *in vitro* BBB was formed by hCMEC or bEnd3 monolayer on the Transwell filter.

Similar to Cancel et al. (2018), DCS was applied to the *in vitro* BBB generated on the Transwell filter by a pair of Ag/AgCl electrodes. The anode, a 4 mm × 1 mm disk (A-M systems, Sequim, WA), was placed on the upper chamber positioned 7 mm above the *in vitro* BBB. The cathode, a 15 mm x 1 mm disk, was placed in the bottom chamber positioned 7 mm below the *in vitro* BBB (Figure 1). A direct current stimulator (model 1300-A, Soterix Medical Inc., New York) was used to deliver a constant 0.1–1 mA/cm^2^ across the *in vitro* BBB for 5 or 10 min. The current level ramped up in ∼30 s to the treatment level and ramped down to zero in ∼ 30 s. This ramping up/down time was excluded from the duration time. The dosage/duration for the *in vitro* experiment was designed to match the typical dosage/duration for the tDCS application in patients with neuronal disorders as well as in the *in vivo* rat experiments (Brunoni et al., 2012; Marceglia et al., 2016; Jackson et al., 2017; Cancel et al., 2018; Shin et al., 2020).

#### Determination of the Blood-Brain Barrier Solute Permeability *in vitro*

The solvent for the permeability measurement was Ringer solution containing 10 mg/mL BSA (Yuan et al., 2010a). At the beginning of the permeability measurement, the upper chamber of the Transwell filter was added with 0.5 mL 10 μM fluorescence solution in 10 mg/mL BSA Ringer while the lower chamber was filled with 1.5 mL blank 10 mg/mL BSA Ringer. The samples of 50 μL were collected every 10 min for 90 min from the lower chamber and were then replaced with the same amount of blank 10 mg/mL BSA Ringer. The fluorescence concentration (intensity) in the samples was determined by SpectraMax M5 microplate reader (Molecule devices, San Jose, CA). The fluorescently labeled solute permeability of the *in vitro* BBB generated on the Transwell filter was calculated by:


$$\text{P}=\frac{\frac{△C_{L}}{△t}\times V_{L}}{C_{U}\times S}$$

where (ΔC_*L*_)/Δt is the increase rate of the fluorescence concentration in the lower chamber, C_*U*_ is the fluorescence concentration in the upper chamber, V_*L*_ is the solution volume in the lower chamber, and S is the surface area of the filter. The permeability P of the blank filter was measured separately and subtracted from the measurement of the total system to obtain the P of the *in vitro* BBB. Since it takes 90 min for the solute permeability measurement, to prevent the change or recovery of the BBB permeability post DCS, we fixed the monolayer right after DCS before measuring its permeability. We tested that the fixation does not affect the monolayer permeability (Supplementary Figure 1).

#### Immunostaining for Endothelial Glycocalyx and Tight Junction ZO-1

EG: Since heparan sulfate (HS) is the most abundant glycosaminoglycan (GAG) of the EG (Sarrazin et al., 2011; Fu and Tarbell, 2013; Zeng et al., 2018), we quantified HS to represent the EG by using the method in Li et al. (2010b); Fan and Fu (2016), Zullo et al. (2016), and Fan et al. (2019). We also labeled another EG component, hyaluronic acid (HA) at hCMEC monolayer (Fan et al., 2019). The hCMEC/bEnd3 monolayer was first washed three times with 10 mg/mL BSA in Dulbecco’s Phosphate-Buffered Saline (DPBS, Corning, Corning, NY) and then fixed with 2% paraformaldehyde (Polyscience, Warrington, PA) and 0.1% glutaraldehyde (Sigma-Aldrich) for 20 min at RT. It was blocked with 2% NGS for 30 min at RT, and incubated with a monoclonal primary antibody to HS (1:100; 10E4 epitope; Amsbio, Cambridge, MA) or biotinylated hyaluronic acid binding protein (50 μg/mL, Amsbio) for hCMEC only at 4 C overnight. After washing three times with DPBS, the cells were incubated for 1 h at RT with AF488 conjugated secondary antibody to HS (1:200, Invitrogen, Thermo Fisher Scientific, Waltham, MA) or AF488 conjugated anti-biotin (1:200, Jackson ImmunoResearch, West Grove, PA) for hCMEC only. The hCMEC/bEnd3 monolayer was washed three times with DPBS, followed with DAPI staining and made into slides for later observation.

Tight junction (ZO-1): The method for labeling ZO-1 of bEnd3 monolayer was described in Li et al. (2010a); Yuan et al. (2010a), and Fan and Fu (2016). The monolayer was washed three times with DPBS, fixed with 1% paraformaldehyde for 10 min, permeabilized with 0.2% Triton X-100 (Sigma-Aldrich, St. Louis, MO) in DPBS for 10 min and blocked with 10% BSA and 0.1% Triton X-100 in DPBS for 1 h in RT. After washing with DPBS, the monolayer was incubated with a primary ZO-1 polyclonal antibody (1:100, 40–2200, Invitrogen, Thermo Fisher Scientific, Waltham, MA) at 4^*o*^C overnight. After three times washed with DPBS, the monolayer was incubated in Alexa Fluor 488 conjugated goat anti-rabbit antibody (1:200, Invitrogen, Thermo Fisher Scientific, Waltham, MA) for 1 h at RT. The monolayer was then washed three times with DPBS, followed with the DAPI staining and made into slides for later observation.

#### Confocal Microscopy and Quantification of Endothelial Glycocalyx and ZO-1

All the samples were imaged by Zeiss LSM 800 confocal laser scanning microscope with 40x oil immersion objective lens (NA = 1.30). For imaging ZO-1, three fields of 160 μm x 160 μm (2,048 × 2,048) were randomly chosen for each sample, and captured as a z-stack of 50–60 images with a z-step of 0.2 μm for two channels (AF488 and DAPI). For EG imaging, three fields of 320 μm by 320 μm (2,048 × 2,048) for each sample were captured as a z-stack of 30–40 images with a z-step of 0.32 μm. Image projection and intensity quantification for EG and ZO-1 were performed by Zeiss ZEN and NIH ImageJ (Fan and Fu, 2016).

#### Data Analysis and Statistics

Data were presented as means ± SE (standard error) unless otherwise specified. Statistical analysis was performed by a two-way (time and different treatment) ANOVA in Prism 8.0. Kurtosis analysis was used to compare the ZO-1 distribution profiles under various conditions. Significance was assumed for probability level *p* < 0.05. For *in vivo* experiments, sample size *n* = 6 at each time point for each treatment, while for *in vitro* experiments, *n* ≥ 6 samples for permeability and TEER, *n* = 3 samples for EG and ZO-1, correspondingly.

## Results

### Effects of Direct Current Stimulation on the Blood-Brain Barrier Permeability (P) to Charged Solutes *in vivo* and *in vitro*

**FIGURE 2:**
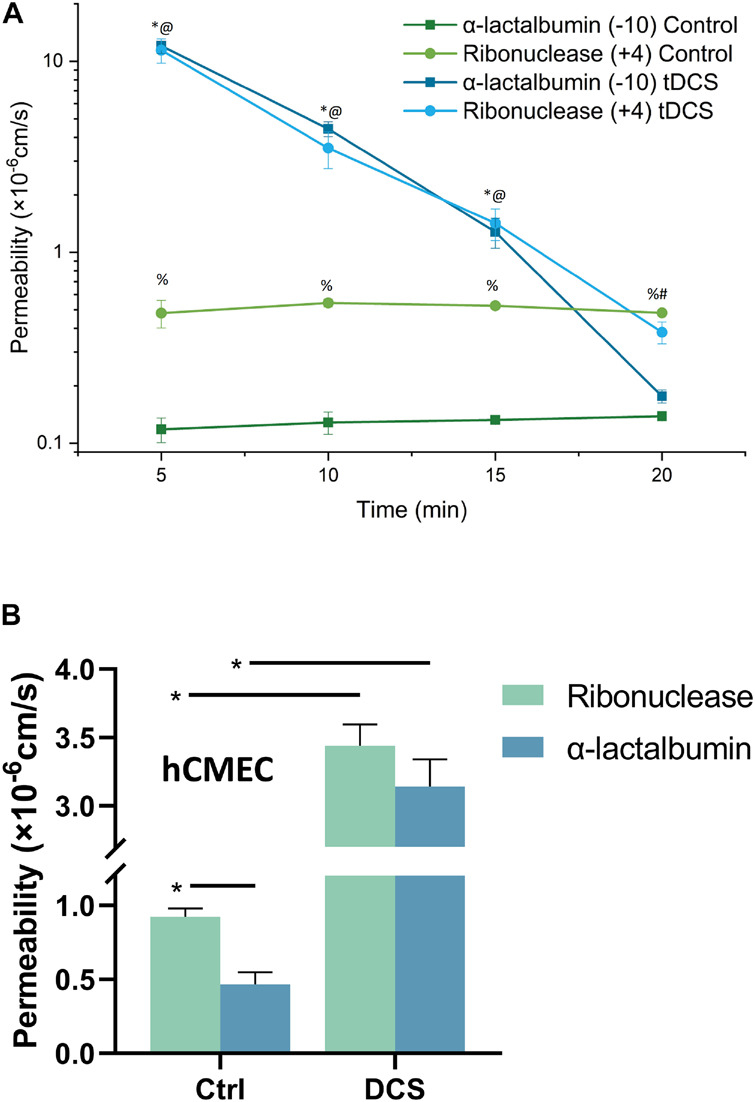
Effects of DCS on the BBB permeability (P) to charged solutes with similar size but opposite charge. **(A)** Comparison of *in vivo* BBB P to FITC-ribonuclease (+4) and that to FITC-α-lactalbumin (–10) under control and 5, 10, 15, 20 min after 20 min 1 mA (8 mA/cm^2^) -tDCS treatment. *,^@^*p* < 0.05, compared with the corresponding controls at the same time; ^%^*p* < 0.05 comparing P to FITC-ribonuclease with that to FITC-α-lactalbumin under control; ^#^*p* < 0.05 comparing P to FITC-ribonuclease and that to FITC-α-lactalbumin 20 min post tDCS; *n* = 6 for each case. **(B)** Comparison of *in vitro* BBB P to FITC-ribonuclease and that to FITC-α-lactalbumin under control and after 10 min 1 mA/cm^2^-DCS treatment. *p < 0.05. The *in vitro* BBB was formed by hCMEC monolayer. *n* = 7 for the control of ribonuclease, *n* = 6 for all other cases.

Since in the structural components of the BBB, only EG and ECM carry negative charge (Yuan et al., 2010b; Li and Fu, 2011; Alphonsus and Rodseth, 2014; Yoon et al., 2017; Kutuzov et al., 2018), we measured the BBB permeability (P) to FITC-ribonuclease and FITC-α-lactalbumin with the same size (Stokes radius ≈ 2.0 nm) but opposite charge *in vivo* after tDCS treatment. If EG and ECM are disrupted by tDCS, the P to these solutes would become the same. Figure 2A demonstrates that under control, the P to the positively charged FITC-ribonuclease (+4) is ∼4-fold that to FITC-α-lactalbumin (−10), indicating that the BBB carrying negative charge under control condition. However, at 5, 10,15 min post tDCS, the P to both solutes significantly increase from their respective controls, but no difference between P to positively charged FITC-ribonuclease and that to negatively charged FITC-α-lactalbumin (*p* > 0.3). The results imply that the charged components of the BBB, EG and ECM, are disrupted by tDCS. At 20 min post tDCS, P to both charged solutes return to their control values, indicating the recovery of EG and ECM and other structural components of the BBB. This is the same as for the P to the neutral solutes reported in Shin et al. (2020).

The above *in vivo* BBB P data to the charged solutes with the same size, and the prediction from a mathematical model for the BBB (Li et al., 2010b; Shin et al., 2020), all suggest that the EG and ECM are temporally disrupted by tDCS, but it is very hard to detect this transient disruption *in vivo*. To directly show that DCS can disrupt EG, we generated an *in vitro* BBB model by culturing a hCMEC monolayer on a Transwell filter with 3 μm pores. For validation, we measured P of this *in vitro* BBB to FITC-ribonuclease and FITC-α-lactalbumin under control and after 10 min treatment with 1 mA/cm^2^ DCS, equivalent dose/duration as those for *in vivo* tDCS (Cancel et al., 2018; Shin et al., 2020). Figure 2B shows that P of *in vitro* BBB to FITC-ribonuclease is ∼2-fold that to FITC-α-lactalbumin, half of the fold as *in vivo*. This is reasonable since no astrocytes in the *in vitro* BBB and ECM is negligible. After DCS treatment, P to these oppositely charged solutes increase from their respective controls but there is no difference between their P right after DCS treatment (*p* = 0.32). The *in vitro* results conform to the *in vivo* data. We thus used this *in vitro* BBB for the effects of DCS on EG.

### Dose Effects of Direct Current Stimulation on *in vitro* Blood-Brain Barrier Transendothelial Electrical Resistance and Permeability to Dextran-70k

**FIGURE 3:**
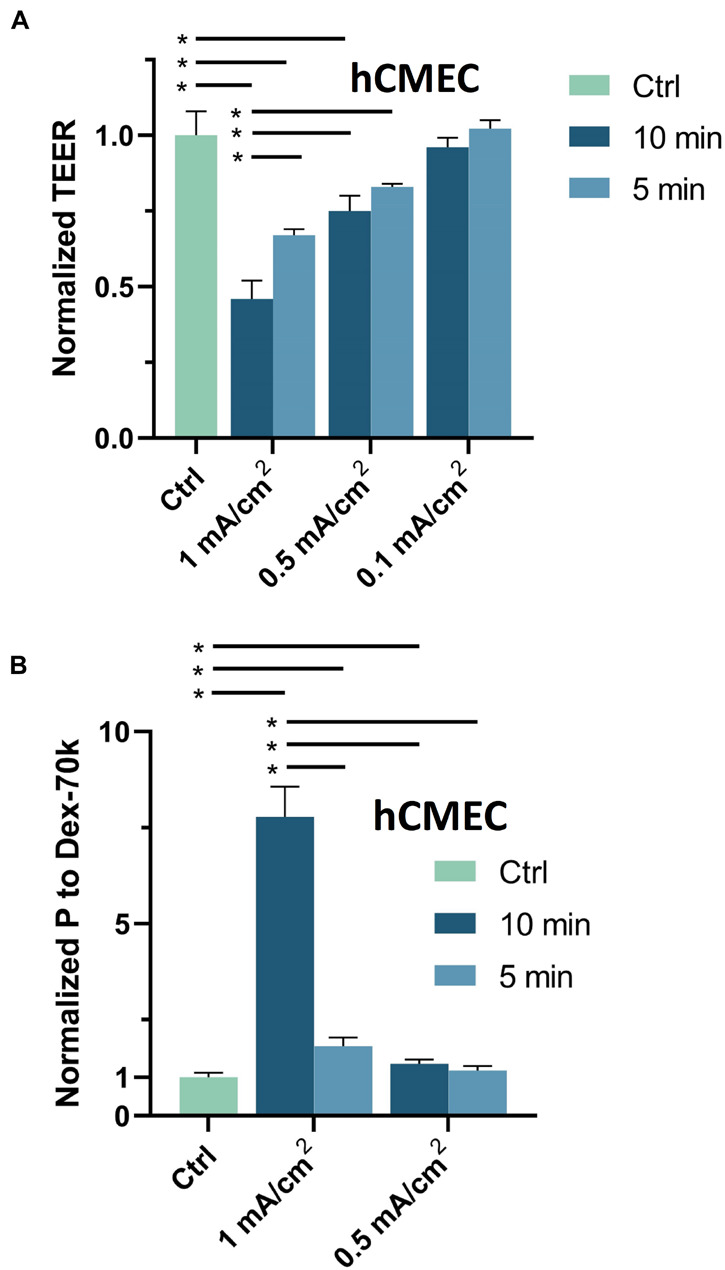
Effects of DCS strength on *in vitro* BBB TEER and permeability. **(A)** Normalized TEER and **(B)** normalized permeability to Dex-70K under control and after 5 min or 10 min treatments with 0.1, 0.5, or 1 mA/cm^2^ DCS. *p < 0.05. The *in vitro* BBB was formed by hCMEC monolayer.

The DCS dosage and duration are important factors in controlling the BBB-disruption levels, we applied 0.1, 0.5 and 1 mA/cm^2^ DCS with duration 5 and 10 min to the *in vitro* BBB to test their effects on the BBB TEER and P to Dex-70k. These dosages and durations were based on the prior studies in rats and humans (Marceglia et al., 2016; Jackson et al., 2017; Cancel et al., 2018; Shin et al., 2020). Figure 3A shows that TEER significantly decreased to 0.46 ± 0.06, 0.67 ± 0.02, 0.75 ± 0.05, 0.83 ± 0.01 of the control, after 1 mA/cm^2^–10 min, 1 mA/cm^2^–5 min, 0.5 mA/cm^2^–10 min and 0.5 mA/cm^2^–5 min DCS treatments, respectively. There were no significant changes in TEER after treatments with 0.1 mA/cm^2^–10 min and 0.1 mA/cm^2^–5 min. The control TEER of hCMEC monolayers is 123.2 ± 2.6 (range 96–134) Ωcm^2^. Correspondingly, Figure 3B demonstrates that P to Dex-70k significantly increased to 7.78 ± 0.78, 1.81 ± 0.23, 1.35 ± 0.11 of the control after 1 mA/cm^2^–10 min, 1 mA/cm^2^–5 min, 0.5 mA/cm^2^–10 min DCS treatments, but no significant change after 0.5 mA/cm^2^–5 min treatment. The control P to Dex-70k is 2.2 ± 0.26 (range 1.4–3.6) × 10^–7^ cm/s. Interestingly, much larger increase in P to Dex-70k occurs at 1 mA/cm^2^-10 min treatment. This is likely due to the significant disruption in the EG of the *in vitro* BBB since EG provides much larger resistance to large solutes such as Dex-70k (Fu and Shen, 2003; Fu et al., 2005; Yuan et al., 2010a; Kutuzov et al., 2018).

### Disruption of Endothelial Glycocalyx on *in vitro* Blood-Brain Barrier by Direct Current Stimulation

**FIGURE 4:**
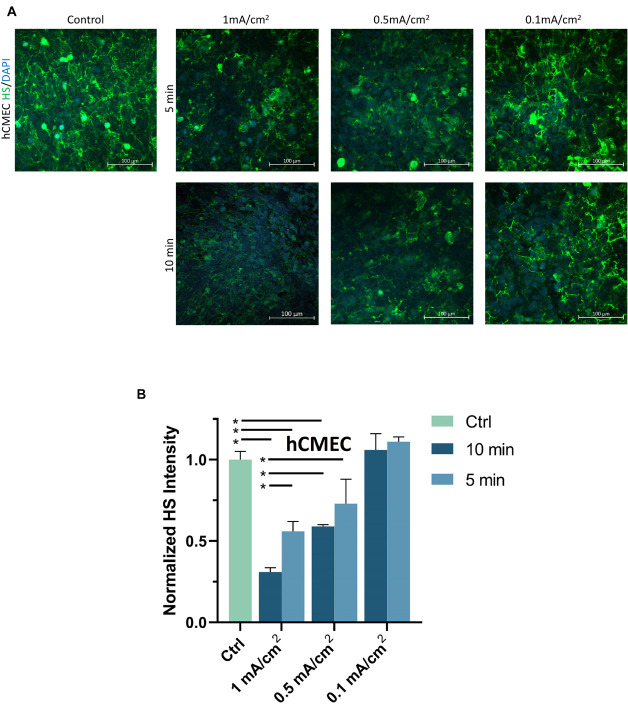
Effects of DCS strength on HS of endothelial glycocalyx (EG) of *in vitro* BBB formed by hCMEC monolayer. **(A)** Confocal images of heparan sulfate (HS) of EG on *in vitro* BBB and **(B)** comparison of HS intensity under control and after 5 min or 10 min treatments with 0.1, 0.5, or 1 mA/cm^2^ DCS. *p < 0.05.

**FIGURE 5:**
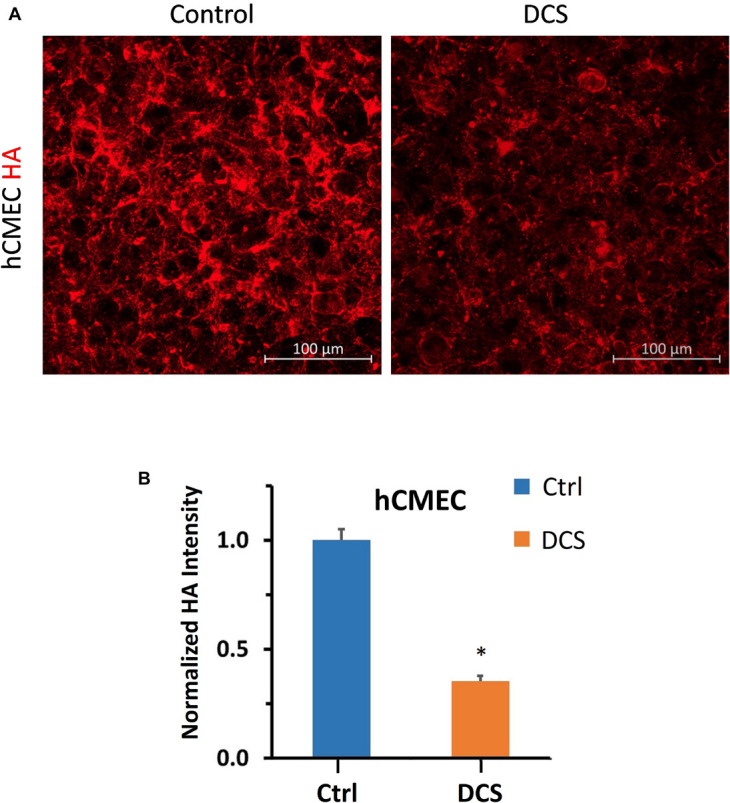
Effects of DCS on HA of endothelial glycocalyx (EG) of *in vitro* BBB formed by hCMEC monolayer. **(A)** Confocal images of hyaluronic acid (HA) of EG on *in vitro* BBB and **(B)** comparison of HA intensity under control and after 10 min treatment with 1 mA/cm^2^ DCS. *p < 0.05.

To investigate the structural mechanism by which DCS decreases BBB TEER but increases P to Dex-70k, we first quantified EG, or heparan sulfate (HS), the most abundant GAG in EG, before and after DCS treatments. Figure 4A demonstrates the confocal images of HS (green) on the hCMEC monolayer under control and after 1 mA/cm^2^–10 min, 1 mA/cm^2^–5 min, 0.5 mA/cm^2^–10 min, 0.5 mA/cm^2^–5 min, 0.1 mA/cm^2^–10 min and 0.1 mA/cm^2^–5 min DCS treatments. Correspondingly, Figure 4B shows that the total intensity of HS decreased significantly to 0.31 ± 0.03, 0.56 ± 0.06, 0.59 ± 0.01 of the control after 1 mA/cm^2^–10 min, 1 mA/cm^2^–5 min and 0.5 mA/cm^2^–10 min treatments; but insignificantly to 0.73 ± 0.14, 1.06 ± 0.1, and 1.11 ± 0.03 of the control after 0.5 mA/cm^2^–5 min, 0.1 mA/cm^2^–10 min and 0.1 mA/cm^2^–5 min DCS treatments, respectively. The effect of DCS on another EG component, hyaluronic acid (HA) of hCMEC monolayers is shown in Figure 5. The intensity of HA decreased to 0.35 ± 0.03 after treatment of 1 mA/cm^2^ DCS for 10 min, similar to the effect of DCS on HS. DCS disrupts EG of the BBB at the dose/duration which can significantly increase P to Dex-70k and decrease TEER.

**FIGURE 6:**
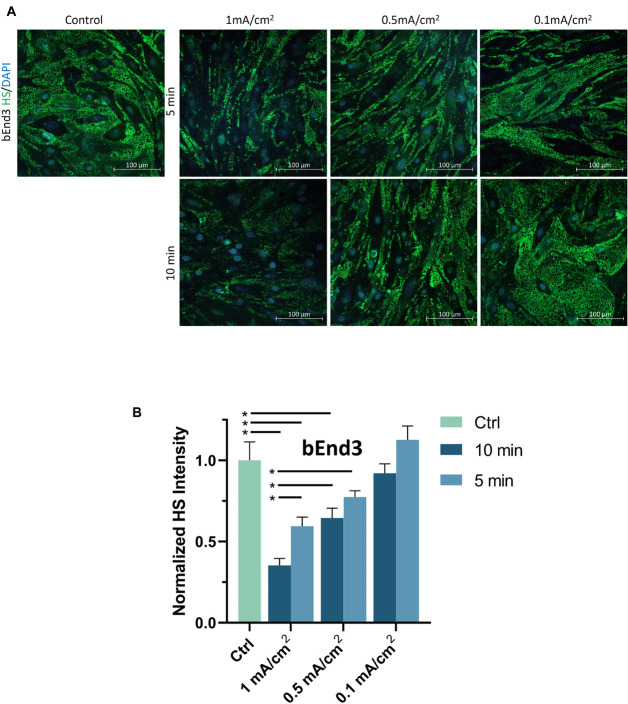
Effects of DCS strength on HS of endothelial glycocalyx (EG) of *in vitro* BBB formed by bEnd3 monolayer. **(A)** Confocal images of heparan sulfate (HS) of EG on *in vitro* BBB and **(B)** comparison of HS intensity under control and after 5 min or 10 min treatments with 0.1, 0.5, or 1 mA/cm^2^ DCS. *p < 0.05.

To examine the effect of DCS on the EG of bEnd3 monolayers, we quantified the HS intensity of bEnd3 after the treatment of DCS with the same levels as for hCMEC monolayers. Figure 6A demonstrates the confocal images of HS on the bEnd3 monolayer under control and after 1 mA/cm^2^–10 min, 1 mA/cm^2^–5 min, 0.5 mA/cm^2^–10 min, 0.5 mA/cm^2^–5 min, 0.1 mA/cm^2^–10 min and 0.1 mA/cm^2^–5 min DCS treatments. Figure 6B shows that the total intensity of HS decreased significantly to 0.35 ± 0.04, 0.59 ± 0.05, 0.65 ± 0.06 of the control after 1 mA/cm^2^–10 min, 1 mA/cm^2^–5 min and 0.5 mA/cm^2^–10 min treatments; but insignificantly to 0.77 ± 0.04, 0.92 ± 0.06, and 1.13 ± 0.08 of the control after 0.5 mA/cm^2^–5 min, 0.1 mA/cm^2^–10 min and 0.1 mA/cm^2^–5 min DCS treatments, respectively. The pattern of DCS effects on HS of bEnd3 monolayers is the same as that on HS of hCMEC monolayers.

### Disruption of Tight Junctions of *in vitro* Blood-Brain Barrier by Direct Current Stimulation

**FIGURE 7:**
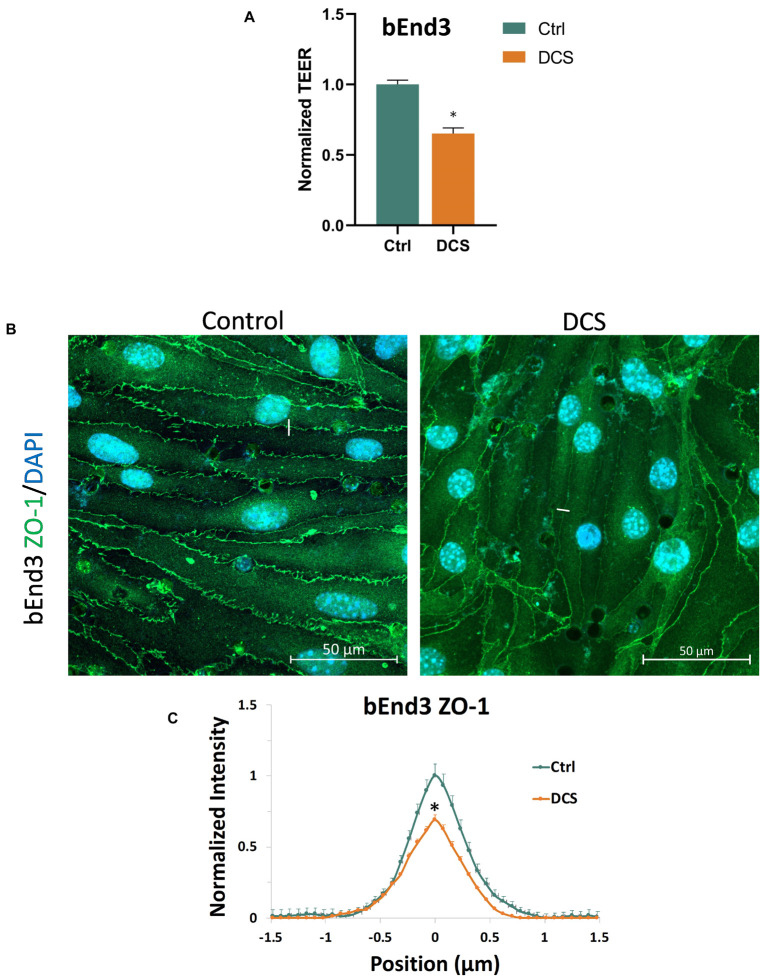
Effects of DCS on TEER and tight junction protein ZO-1 of *in vitro* BBB formed by bEnd3 monolayer. **(A)** Normalized TEER, **(B)** confocal images of ZO-1 of *in vitro* BBB, and **(C)** comparison of the intensity profiles of ZO-1 along a ∼3 μm line perpendicular to the EC junctions (white lines in the confocal images) under control and after 10 min treatment with 1 mA/cm^2^ DCS. The peak intensity of ZO-1 from the control was used for the normalization. *n* = 120 profiles for junctions between 30 ECs were averaged for each plot. ^∗^*p* < 0.05.

To investigate another structural change, the junction proteins between ECs, by the DCS to increase BBB permeability, we quantified the tight junction, ZO-1 of the *in vitro* BBB formed by bEnd3 monolayer since no good labeling was found for the junction proteins of hCMEC monolayer (Supplementary Figure 2). We first measured the effect of DCS on the TEER of bEnd3 monolayers. Figure 7A shows that after 1 mA/cm^2^–10 min DCS treatment, TEER of bEnd3 monolayers significantly decreased to 0.65 ± 0.04. The control TEER of bEnd3 monolayer is 140.3 ± 3.5 (range 127–160) Ωcm^2^. Figure 7B demonstrates the confocal images of ZO-1 (green) of bEnd3 monolayer under control and after 1 mA/cm^2^–10 min DCS treatment. Correspondingly, Figure 7C compares the ZO-1 concentration (intensity) distribution under control and after DCS treatment. Figure 7C shows that ZO-1 was significantly disrupted after 1 mA/cm^2^–10 min DCS treatment.

### Effects of Nitric Oxide Synthase Inhibition, Nitric Oxide and Combined Effects of Nitric Oxide Synthase Inhibition and Direct Current Stimulation on *in vitro* Blood-Brain Barrier Permeability

**FIGURE 8:**
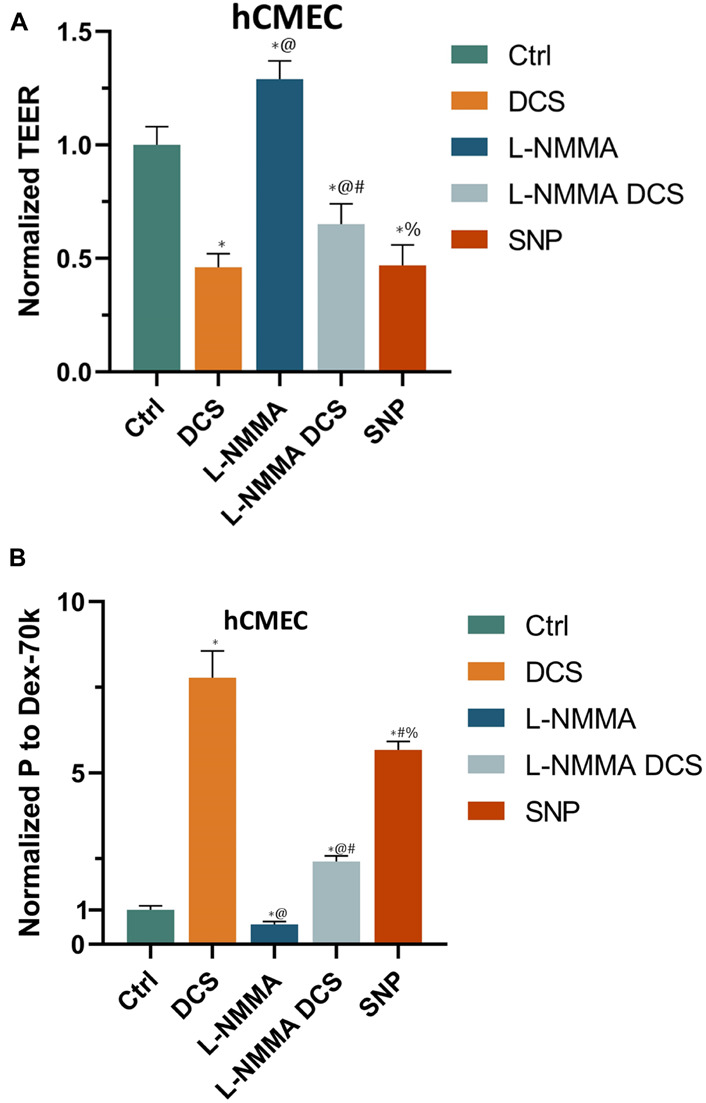
Effects of a NOS inhibitor (L-NMMA) and a NO donor, sodium nitroprusside (SNP) on *in vitro* BBB TEER and permeability. **(A)** Normalized TEER and **(B)** normalized permeability to Dex-70K under control and after treatment of 10 min 1 mA/cm^2^ DCS (DCS), or treatment of 60 min 1 mM L-NMMA, or pretreatment of 60 min 1 mM L-NMMA and treatment of 10 min 1 mA/cm^2^ DCS (L-NMMA DCS), or treatment of 30 min 300 μM SNP (SNP). *n* = 6 samples for each case in **(A,B)**. *p < 0.05, compared with the control, ^@^*p* < 0.05, compared with DCS, ^#^*p* < 0.05, comparing L-NMMA DCS and SNP with L-NMMA, ^%^*p* < 0.05, comparing SNP with L-NMMA DCS. The *in vitro* BBB was formed by hCMEC monolayer.

Prior studies have reported that activation of NOS by cytokines or inflammatory agents enhances endothelial NO release to increase microvascular permeability (Duran et al., 2010; Shi et al., 2014a). Our recent *in vivo* study also showed that tDCS-induced BBB permeability increase is NO dependent (Shin et al., 2020). To test if DCS-induced BBB permeability increase is also NO dependent in the *in vitro* BBB, we measured TEER and P to Dex-70k of hCMEC monolayers after treatment of 1 mA/cm^2^ DCS for 10 min, after treatment of a NOS inhibitor, L-NMMA, for 60 min, and pretreatment with L-NMMA before 1 mA/cm^2^-10 min DCS treatment. Inhibition of NOS by 1 mA L-NMMA for 60 min significantly increased TEER to 1.29-fold (or 129%) that of the control (Figure 8A), and decreased P to Dex-70k to 0.58 (Figure 8B) of the control, respectively. Pretreatment of 1 mM L-NMMA for 60 min significantly diminished the effects of DCS on both TEER and P to Dex-70k, like what observed in the *in vivo* study (Shin et al., 2020). We also tested the effect of a NO donor, SNP, on the permeability of the *in vitro* BBB. Treatment of 300 μM SNP for 30 min significantly decreased TEER to 0.47 (Figure 8A) and increased P to Dex-70k to 5.67-fold (Figure 8B) that of the corresponding controls. Treatment with 300 μM SNP for 30 min has a similar effect on the TEER compared to the treatment of 1 mA/cm^2^ DCS for 10 min but its effect on P to Dex-70k is smaller than that of DCS.

### Disruption of Endothelial Glycocalyx by Direct Current Stimulation Is Nitric Oxide Dependent

**FIGURE 9:**
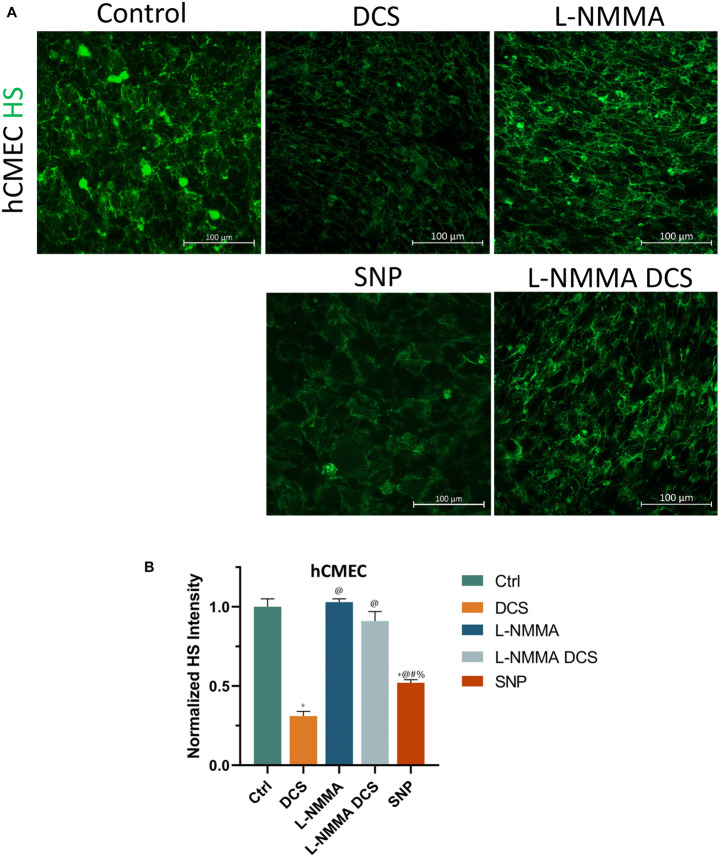
Effects of a NOS inhibitor (L-NMMA) and a NO donor, sodium nitroprusside (SNP) on endothelial glycocalyx (EG) of *in vitro* BBB formed by hCMEC monolayer. **(A)** Confocal images of heparan sulfate (HS) of EG on *in vitro* BBB and **(B)** comparison of HS intensity under control and after treatment of 10 min 1 mA/cm^2^ DCS (DCS), or treatment of 60 min 1 mM L-NMMA, or pretreatment of 60 min 1 mM L-NMMA and treatment of 10 min 1 mA/cm^2^ DCS (L-NMMA DCS), or treatment of 30 min 300 μM SNP (SNP). *n* = 3 samples for each case in **(B)**. *p < 0.05, compared with the control, ^@^*p* < 0.05, compared with DCS, ^#^*p* < 0.05, comparing L-NMMA DCS and SNP with L-NMMA, ^%^*p* < 0.05, comparing SNP with L-NMMA DCS.

To investigate if EG disruption by DCS is NO dependent, we quantified the EG of hCMEC monolayers under treatments of NO inhibition, NO and pretreatment of NOS inhibitor before DCS. The doses and durations of the treatments are the same as in Figure 8. Figure 9A demonstrates the confocal images of HS (green) on the hCMEC monolayer under control, DCS, SNP, NO inhibition by L-NMMA, and pretreatment with L-NMMA before DCS. Figure 9B compares the HS intensity under various treatments. L-NMMA alone has no influence on EG of *in vitro* BBB. Pretreatment with L-NMMA before DCS seems to protect the EG from DCS disruption. SNP greatly degrades the HS to 0.52 of the control, which has slightly smaller effect than that by DCS (0.31). The results indicate that disruption of EG by DCS is NO dependent.

### Disruption of Tight Junctions by Direct Current Stimulation Is Nitric Oxide Dependent

**FIGURE 10:**
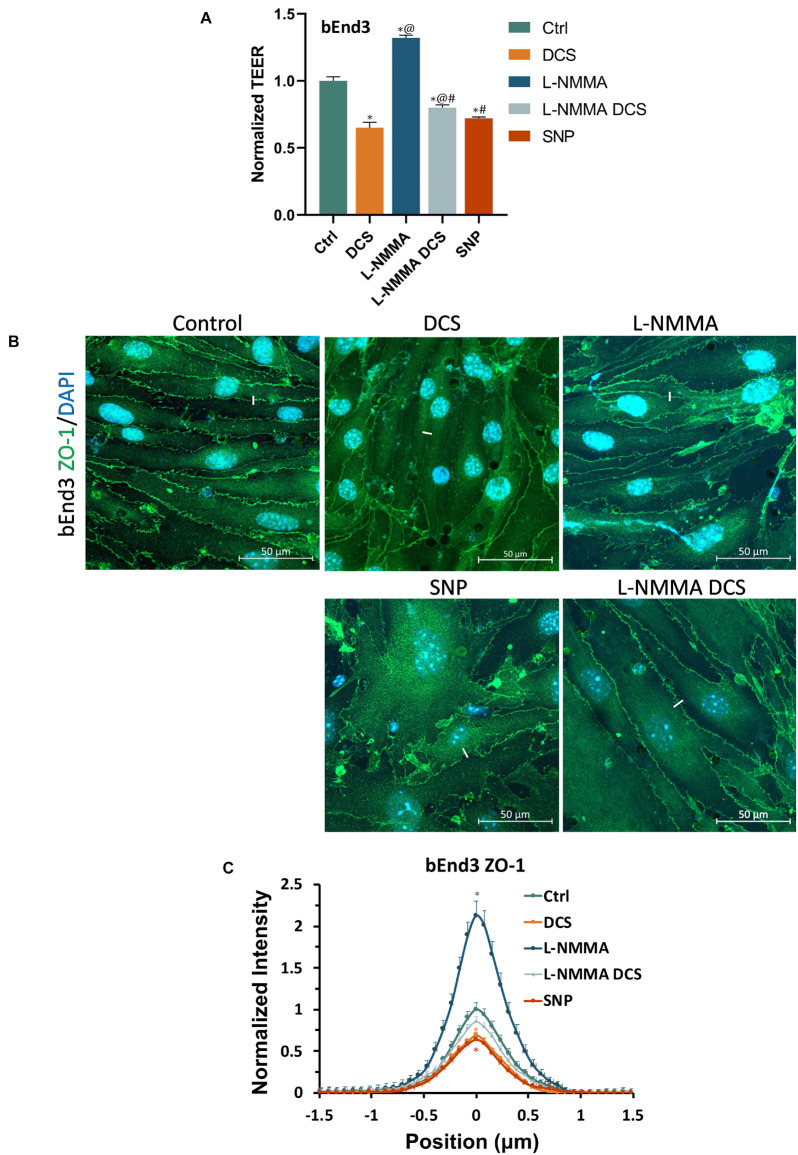
Effects of a NOS inhibitor (L-NMMA) and a NO donor, sodium nitroprusside (SNP) on TEER and tight junction protein ZO-1 of *in vitro* BBB formed by bEnd3 monolayer. **(A)** Normalized TEER, **(B)** confocal images of ZO-1 of *in vitro* BBB, and **(C)** comparison of the intensity profiles of ZO-1 along a ∼3 μm line perpendicular to the EC junctions (white lines in the confocal images) under control and after treatment of 10 min 1 mA/cm^2^ DCS (DCS), or treatment of 60 min 1 mM L-NMMA, or pretreatment of 60 min 1 mM L-NMMA and treatment of 10 min 1 mA/cm^2^ DCS (L-NMMA DCS), or treatment of 30 min 300 μM SNP (SNP). *n* = 6 samples for each case in **(A)** and *n* = 3 samples for each case in **(C)**. *p < 0.05, compared with the control, ^@^*p* < 0.05, compared with DCS, ^#^*p* < 0.05, comparing L-NMMA DCS and SNP with L-NMMA, ^%^*p* < 0.05, comparing SNP with L-NMMA DCS.

To further investigate if tight junction disruption by DCS is NO dependent, we quantified ZO-1 of bEnd3 monolayers under treatments of NO inhibition, NO and pretreatment of NOS inhibitor before DCS. The doses and durations of the treatments are the same as in Figure 8. Like what in Figure 8A, we measured the TEER of bEnd3 monolayer under these treatments, which are shown in Figure 10A. The effects of these treatments on TEER of bEnd3 monolayer are the same as those on TEER of hCMEC monolayer. Figure 10 demonstrates the confocal images of ZO-1 (green) on the bEnd3 monolayer under control, DCS, SNP, NO inhibition by L-NMMA, and pretreatment with L-NMMA before DCS. Figure 10C compares the ZO-1 intensity distribution profiles under various treatments. In contrast to that on EG, L-NMMA alone significantly enhances the ZO-1 intensity to ∼2-fold that of the control. Pretreatment with L-NMMA before DCS partially abolishes the ZO-1 disruption by DCS. SNP greatly disrupts ZO-1 to 0.64 of the control, the same as that by DCS (0.69). The results indicate that disruption of ZO-1 by DCS is NO dependent.

## Discussion

Prior studies have shown the direct effects of electrical stimulation on endothelial cells (ECs), including re-orientation and secretion of vascular endothelial growth factors (VEGFs) (Bai et al., 2011) and NO (Trivedi et al., 2013). Our current study shows the direct effects of electrical stimulation (or DCS) on the *in vitro* BBB formed by the confluent EC monolayer with the comparable permeability to that of cerebral microvessels in rats (Shi et al., 2014b; Shin et al., 2020). The decreased TEER and increased P to Dex-70k by DCS found in the current study are consistent with those prior findings *in vitro* since VEGF can enhance BBB permeability *in vivo* and *in vitro* (Shi et al., 2014a; Fan and Fu, 2016), and NO (by a NO donor, SNP) can decrease TEER and increase P to Dex-70k of *in vitro* BBB. Our *in vitro* results are also consistent with those from the prior *in vivo* study that tDCS can increase BBB permeability and this increase is NO dependent. Inhibition of NOS by pretreatment of L-NMMA reduced the BBB permeability increase by tDCS in rats (Shin et al., 2020). Nevertheless, in the *in vivo* setting, it is unclear if L-NMMA inhibits NOS of ECs, or NOS from other types of cells in the brain, or both. The current study on the *in vitro* BBB with only ECs showed that L-NMMA can inhibit NOS of ECs to diminish the increased BBB permeability by DCS, indicating that DCS can directly activate NOS of ECs in the BBB. The released NO from ECs by the DCS can modulate the blood vessel dilation and enhance blood perfusion to the brain, which were reported in humans (Stagg et al., 2013; Wang et al., 2015) and in animals (Mielke et al., 2013).

To investigate the structural mechanisms by which DCS altered BBB permeability, based on their *in vivo* permeability data for small and large solutes, by employing a transport model for the paracellular pathway of the BBB (Li et al., 2010b), Shin et al. (2020) predicted that tDCS increases the BBB permeability by altering the barrier structural components of the BBB, i.e., disrupting the EG, ECM, and tight junctions of ECs, as well as enlarging the width of the inter-endothelial cleft and the width of the BM. In the *in vivo* experiment of the current study, the finding that after tDCS treatment, the BBB permeability to the same sized solutes with opposite charge became identical although their permeability has 4-fold difference in the absence of tDCS, also suggests that the EG and ECM are temporally disrupted by tDCS because only the EG and ECM carry charge in the BBB.

However, it seems very challenging to detect the above structural changes *in vivo* due to transient behavior and nano-scale structures. We thus generated two *in vitro* BBB models, one formed by human cerebral microvascular endothelial cells (hCMEC) monolayer and another by mouse brain microvascular endothelial cells (bEnd3) monolayer. The generated *in vitro* BBB by both types of ECs has comparable solute permeability to that of rat cerebral microvessels (Yuan et al., 2010a,b; Shi et al., 2014b; Shin et al., 2020). The reason for using two types of ECs is that there was no good labeling for any junction formed on the hCMEC monolayer (Supplementary Figure 2) but good labeling for the tight and adherens junctions especially ZO-1 formed on bEnd3 monolayer (Li et al., 2010a; Yuan et al., 2010a; Fan and Fu, 2016).

The baseline TEER for hCMEC monolayers is 123.2 ± 2.6 Ωcm^2^ and that for bEnd3 monolayers is 140.3 ± 3.5 Ωcm^2^. The higher TEER of bEnd3 monolayers reflects a better tight junction at bEnd3 monolayers than that at hCMEC monolayers (Fu et al., 1994; Sugihara-Seki and Fu, 2005). But the higher permeability of bEnd3 monolayers to Dex-70k, 4.4 × 10^–7^ cm/s (Yuan et al., 2010a), compared to 2.2 × 10^–7^ cm/s of hCMEC monolayers (current study), suggests a fewer EG on bEnd3 monolayers (Fu and Shen, 2003; Sugihara-Seki and Fu, 2005; Yuan et al., 2010a; Kutuzov et al., 2018). By immunostaining the EG of the hCMEC/bEnd3 monolayer formed *in vitro* BBB and the tight junction ZO-1 of the bEnd3 monolayer formed *in vitro* BBB, we found that DCS disrupts the EG and ZO-1 when it is at a strength which significantly decreases the TEER and increases permeability to Dex-70k.

In the *in vivo* study on rats, the increased BBB permeability by the tDCS returned to the control level in 20 min. The recovery of the BBB permeability by the proper strength of tDCS guarantees its safety in clinical applications. Although in the *in vitro* study, we used the equivalent or smaller strength of DCS compared to that used *in vivo*, we did not see the recovery of the BBB permeability after 60 min (Supplementary Figure 3). Under this strength of DCS, there were no visible changes in the monolayer before and after treatment (Supplementary Figure 4). The possible reason is that in the brain, the BBB is a 3-D tube structure formed by ECs and surrounding pericytes and astrocyte foot processes. The tDCS- enlarged inter-cellular gaps by EC contractions can go back to the baseline when the tDCS triggered Ca^2+^ and NO release (Marceglia et al., 2016; Monai et al., 2016) is over. The EG can be reconstructed by the components existing in the circulating blood (Zeng et al., 2018; Weinbaum et al., 2021). The disrupted EG and tight junctions can also be resynthesized by ECs (Zihni et al., 2016; Komarova et al., 2017) in the proper environment. In contrast, the *in vitro* BBB under study is formed by the EC monolayer cultured on a Transwell filter, which is a 2-D structure. When its ECs contract under DCS, the inter-endothelial cleft may not be able to go back to the original due to the attachment of the ECs and the filter membrane. The disrupted EG and tight junctions are also not able to recover due to lack of enough building components in the cell culture medium or lack of the proper environment for the re-synthesization. Although the *in vitro* 2-D BBB model cannot completely mimic the *in vivo* effects of tDCS on the BBB, it provides a convenient and direct measurement (snapshot) on the changes in the structural components of the BBB by DCS.

Cancel et al. (2018) used bEnd3 cells to generate an *in vitro* BBB. They showed that DCS can modulate water permeability of this *in vitro* BBB through electroosmosis but they did not observe the EC tight junction disruption by the DCS with the similar strength as we used. The discrepancy is that they used the Transwell filter with 0.4 μm pores which only accounts for 0.5% of the surface area of the filter, 99.5% of the area is not electrically conductive due to the material used to make the filter. In their set-up, only 0.5% of the EC monolayer received the DCS. Instead, we used the Transwell filter with 3 μm pores which accounts for 14.1% of surface area of the filter, 28.2-fold that of the pore area in the filter used in Cancel et al. (2018).

To further investigate the cellular signaling mechanisms by which DCS disrupts the EG and tight junction to increase the BBB permeability, we treated the *in vitro* BBB with a NOS inhibitor, L-NMMA, and a NO donor, SNP, as well as pretreatment with L-NMMA before DCS. We found that exogenous NO (by SNP) disrupts the EG and ZO-1, the same as DCS behaves. Although inhibition of NOS (to reduce NO release by ECs) by L-NMMA has no effects on the EG under control conditions, it can prevent the EG disruption from the DCS. On the other hand, L-NMMA can stimulate the formation of tight junction ZO-1 under control conditions and the reinforced tight junctions can diminish the tight junction disruption by the DCS. Our current results are consistent with previous studies. A prior study reported that NOS inhibition for 35 min or longer by 1 mM L-NMMA reduced the microvascular permeability of rat mesenteric microvessels to below the baseline value *in vivo* (Zhang et al., 2016). The reduced NO production by NOS inhibition was found to elevate intracellular cAMP levels by inhibiting phosphodiesterase 3 (Surapisitchat et al., 2007). cAMP has been reported to decrease permeability by strengthening the tight junction integrity (Adamson et al., 1998; Spindler et al., 2011; Fu et al., 2015; Fan and Fu, 2016).

Although hCMEC/bEnd3 monolayers may not be the best *in vitro* BBB model, their permeability to Dex-70k, 2.2 × 10^–7^ cm/s for hCMEC (in current study) and 4.4 × 10^–7^ cm/s for bEnd3 (Yuan et al., 2010a) are comparable to the permeability of rat cerebral microvessels measured *in vivo*, 1.1-1.3 × 10^–7^ cm/s (Shi et al., 2014b; Shin et al., 2020). In addition, both monolayers have significant EG (HS) and bEnd3 has a very good tight junction protein ZO-1 expression. Furthermore, the TEER of both monolayers (123.2 Ωcm^2^ for hCMEC and 140.3 Ωcm^2^ for bEnd3) is much higher than the best TEER (∼75 Ωcm^2^) of the mono-culture and co-culture *in vitro* BBB models formed from primary human cells including brain microvascular endothelial cells, astrocytes, pericytes and neurons (Stone et al., 2019). The permeability of hCMEC and bEnd3 monolayers to Dex-70k (2.2 and 4.4 ×10^–7^ cm/s) is much smaller than that of the *in vitro* BBB formed from rat primary brain microvascular endothelial cells (Romero et al., 2003), which is 6.3 ×10^–7^ cm/s. Therefore, the *in vitro* BBB models formed by hCMEC and bEnd3 cell lines are suitable for our purpose to directly visualize the structural changes in the BBB by the DCS, which can only be predicted by a mathematical model from the measured permeability data *in vivo*. Certainly, a better *in vitro* BBB model by using primary cells and co-coculture with the astrocytes/pericytes to achieve better barrier property should be utilized in the future study. In addition, a 3-D circular shaped *in vitro* BBB surrounded by a proper brain tissue mimicking hydrogel with a continuous perfusion system can be generated to simulate the real physiological conditions. Furthermore, to directly measure the effect of tDCS on the BBB ultra-structures, it is expected to develop an *in vivo* detecting technique by utilizing high-resolution multiphoton microscopy (Shi et al., 2014b; Kutuzov et al., 2018) and specific biomarkers for the junction proteins and glycocalyx/ECM, or transgenic animal models with optically detectable junction proteins and glycocalyx/ECM.

## Conclusion

In conclusion, our current study reveals that DCS increases the BBB permeability by disrupting the endothelial glycocalyx and tight junctions of the BBB and the disruption is NO dependent.

## Data Availability Statement

The raw data supporting the conclusions of this article will be made available by the authors, without undue reservation.

## Ethics Statement

The animal study was reviewed and approved by the Animal Care and Use Committee at The City College of the City University of New York.

## Author Contributions

The experiments described here were performed in the Microcirculation Laboratory at The City College of the City University of New York. BF and YX contributed to the conception and design of the work. YX, YL, WK, and BF contributed to acquisition, analysis, and interpretation of data for the work. YX, MB, and BF contributed to drafting and revising the work. All authors approved the final version of the manuscript, agreed to be accountable for all aspects of the work in ensuring that questions related to the accuracy or integrity of any part of the work are appropriately investigated and resolved, designated as authors qualify for authorship, and all those who qualify for authorship are listed.

## Conflict of Interest

The authors declare that the research was conducted in the absence of any commercial or financial relationships that could be construed as a potential conflict of interest.

## Publisher’s Note

All claims expressed in this article are solely those of the authors and do not necessarily represent those of their affiliated organizations, or those of the publisher, the editors and the reviewers. Any product that may be evaluated in this article, or claim that may be made by its manufacturer, is not guaranteed or endorsed by the publisher.

## Supplementary Material

Supplementary Figure 1 Comparison of *in vitro* BBB permeability to Dex-70k measured with and without fixation. The *in vitro* BBB was formed by hCMEC monolayer.

Supplementary Figure 2 Confocal images of ZO-1, claudin-5, occludin and VE-Cadherin at the *in vitro* BBB formed by hCMEC monolayer.

Supplementary Figure 3 Comparison of normalized *in vitro* BBB permeability to Dex-70k under control and 0, 10, 30 and 60 min post 10 min 1 mA/cm^2^ DCS treatment. ^∗^*p* < 0.05. The *in vitro* BBB was formed by hCMEC monolayer.

Supplementary Figure 4 Bright-field images of the bEnd3 monolayer before and after 10 min–1 mA/cm^2^ DCS.
