# Supplementary figures and images for: Direct Current Stimulation Disrupts Endothelial Glycocalyx and Tight Junctions of the Blood-Brain Barrier in vitro
Source: Front Cell Dev Biol. 2021 Sep 28;9:731028. doi: 10.3389/fcell.2021.731028 (PMC8505730; doi:10.3389/fcell.2021.731028)

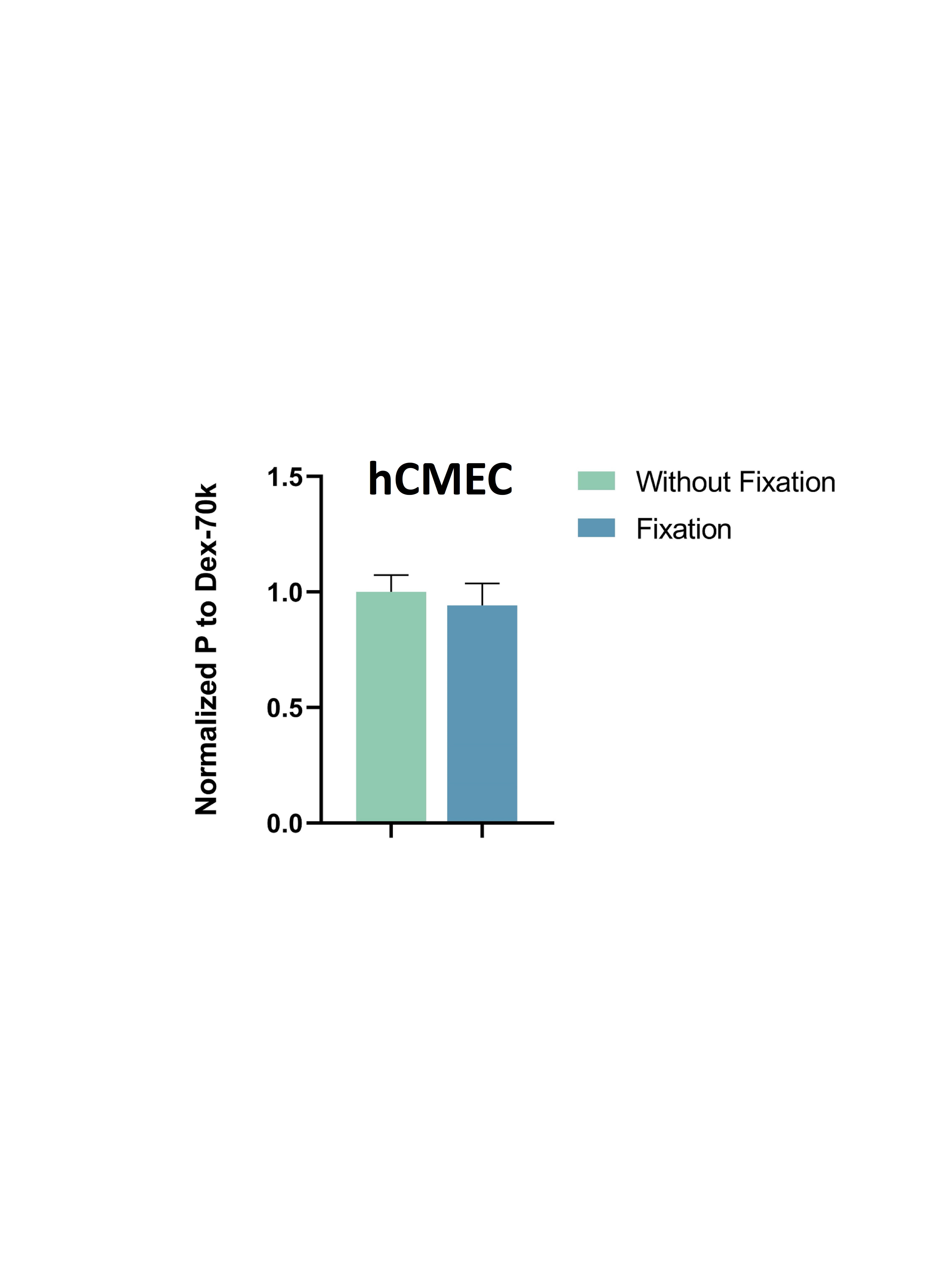

Supplement: Supplementary Figure 1 — Comparison of in vitro BBB permeability to Dex-70k measured with and without fixation. The in vitro BBB was formed by hCMEC monolayer. [file Image_1.tif]

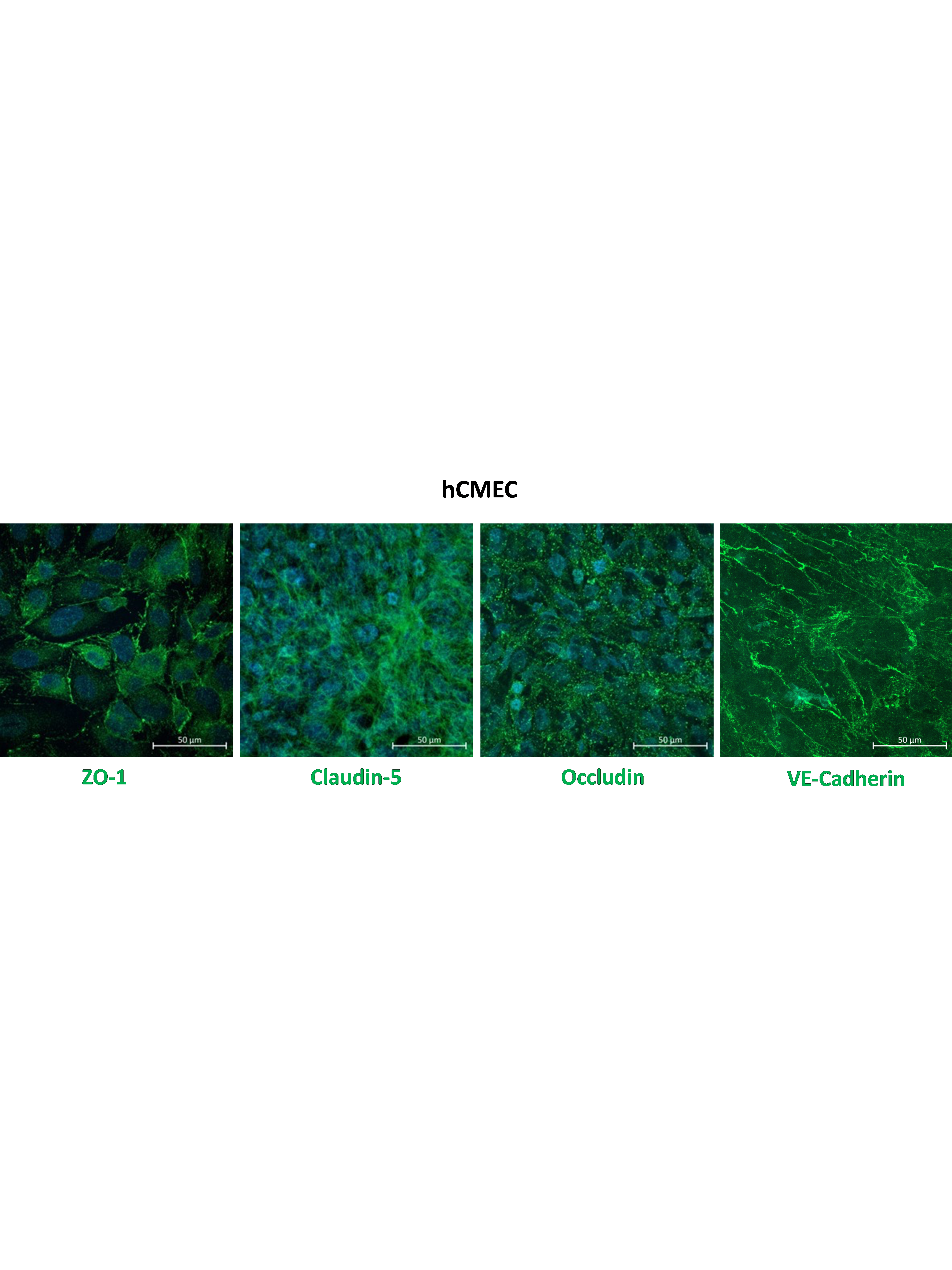

Supplement: Supplementary Figure 2 — Confocal images of ZO-1, claudin-5, occludin and VE-Cadherin at the in vitro BBB formed by hCMEC monolayer. [file Image_2.tif]

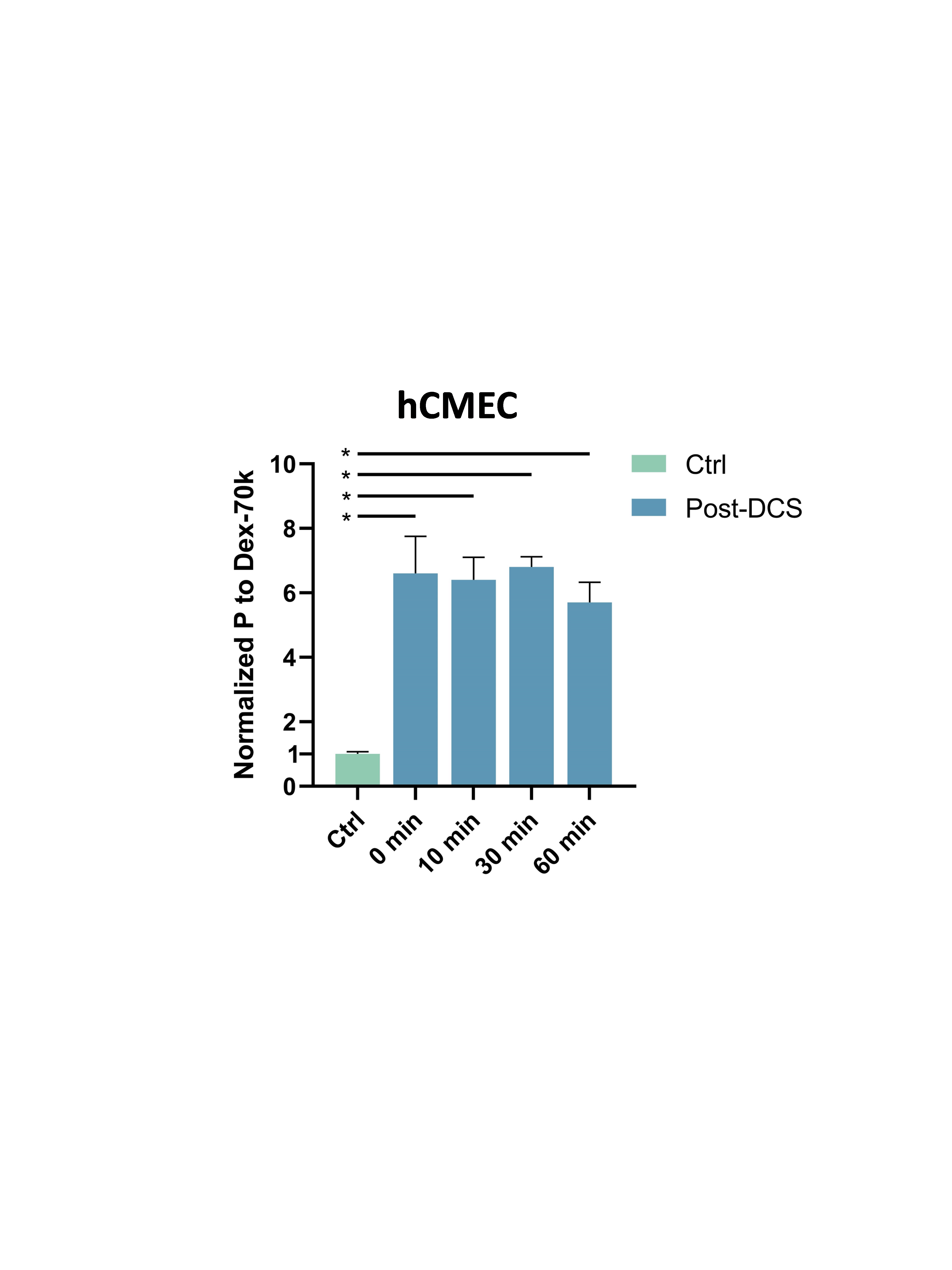

Supplement: Supplementary Figure 3 — Comparison of normalized in vitro BBB permeability to Dex-70k under control and 0, 10, 30 and 60 min post 10 min 1 mA/cm2 DCS treatment. ∗p < 0.05. The in vitro BBB was formed by hCMEC monolayer. [file Image_3.tif]

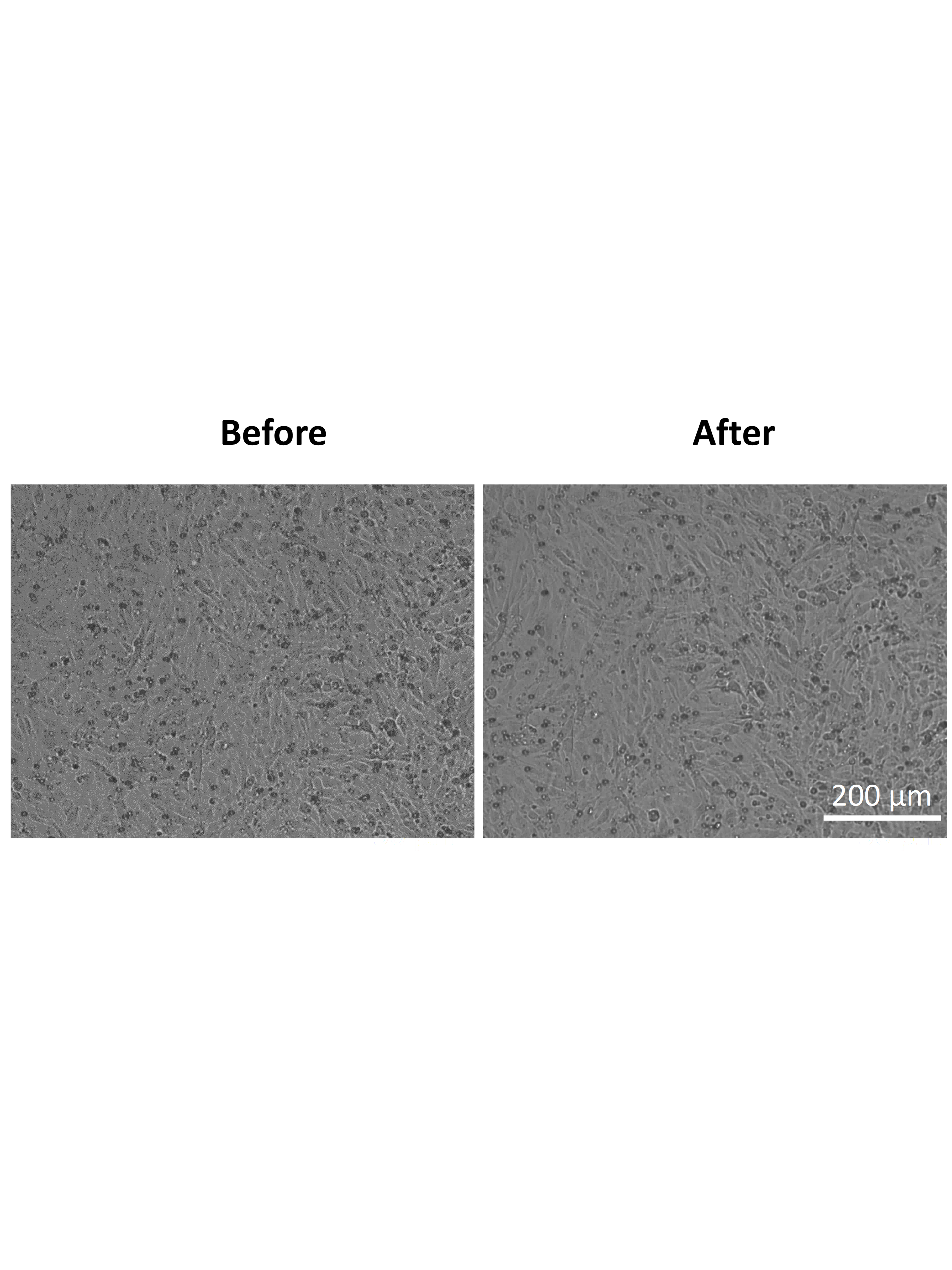

Supplement: Supplementary Figure 4 — Bright-field images of the bEnd3 monolayer before and after 10 min–1 mA/cm2 DCS. [file Image_4.tif]
